# Use of music during vaginal birth and caesarean section: an interprofessional survey

**DOI:** 10.1007/s00404-020-05958-9

**Published:** 2021-01-24

**Authors:** Philip Hepp, Markus Fleisch, Kathrin Hasselbach, Tanja Fehm, Nora K. Schaal

**Affiliations:** 1 University Witten/Herdecke, Landesfrauenklinik, Wuppertal, Germany; 2 University Hospital Augsburg, Frauenklinik, Stenglinstr. 2, 86156 Augsburg, Germany; 3 Department for Cognitive Psychology, Heinrich-Heine-University Düsseldorf, Düsseldorf, Germany; 4 University Hospital Düsseldorf, Frauenklinik, Heinrich-Heine-University Düsseldorf, Düsseldorf, Germany

**Keywords:** Survey, Sound, Obstetrics, Staff, Midwives, Birth

## Abstract

**Purpose:**

Evidence abounds about the beneficial effects of music on patients and healthcare professionals for many medical indications. This study aimed to evaluate the dissemination and use of music in the obstetrical setting.

**Methods:**

Invitations to an online survey were sent to physicians and midwives of all obstetrics departments in Germany. The survey gathered descriptive data as well as information about the personal relation to music and the use of it during vaginal birth (VB) and caesarean section (CS) and whether data about positive or negative effects of music were known to the participant.

**Results:**

In total, there were 293 respondents. The 47% that had the means to play music during CS stated that music was played in 15% of the cases. Most respondents have the means to play music during VB (97%). Music is played in 38% of VB. Regardless of the mode of delivery, music was estimated to be positive for team communication and patient communication. It was also deemed calming and mood lifting on the respondents. Regarding the patient, music during CS and VB was rated as being positive on all scales. Listening to music was recommended more often during VB (66%) than CS (38%).

**Conclusions:**

Although healthcare professionals are mostly aware of the beneficial effects of music in obstetrics, our study shows that music plays a more important role during VB than during CS in Germanys obstetrical wards. There is a lack of equipment to play music in operation theatres where CS take place.

## Introduction

Music is a non-pharmaceutical, cost-effective and easy-to-use tool in clinical practice. There are data for a variety of indications and medical settings in which music has been proved to provide beneficial effects (for review see [1]). In particular, the analgesic and anxiolytic effect makes music particularly useful for obstetricians. Two randomized controlled trials show consistently that it has a strong impact on vaginal birth (VB) by significantly reducing the amount of stress and anxiety perceived by the parturient measured by visual analogue scales during all stages of labour and significantly reducing blood pressure and heart rate [2]. Especially noteworthy, the effect seems to prevail even after delivery where patients of the music intervention group had significantly less usage of pain medication in the first 24 h following delivery [3]. Another prospective, interventional study showed that parturients who listened to music were more likely to deliver spontaneously compared to those who did not [4].

Analogously there is increasing evidence about the advantageous effects of music in the setting of caesarean section (CS). Again several studies showed a reduction in stress and anxiety perceived by the mother during [5] and while preparing for CS [6] with an effect even hours after the music intervention ended.

Besides possible positive effects on patients, also the medical personnel may profit from music in their working environment. In a questionnaire-based study among surgical staff comprising physicians as well as nurses, a majority reported regular use of music as a mean to reduce stress [7]. Another cross-sectional prospective study among healthcare professionals showed that music helps to improve concentration while soothing anxiety of the patient by “producing a sense of familiarity in a strange environment” [8].

Nevertheless, concerning music’s impact on healthcare professionals there are opposing data indicating that music could interfere with communication within medical teams and therefore yields possible detrimental effects on the patient’s treatment and well-being [9]. For instance, one study revealed that the necessity for an interrupted request/reaction chain and therefore the need for a repeated request within a team increase fivefold during a medical procedure if music is present. In this study, every interruption of the request/reaction chain led to a delay of 4–68 s and “increased tensions due to frustration at ineffective communication” [10].

Given the growing body of evidence about possible positive and negative effects of music in the medical setting, it seems worthwhile to evaluate its actual usage in everyday routine. To this end, obstetricians and midwives seem to be an important source of information as the process of CS and VB is always accompanied by them. Furthermore, it seems important to assess their attitude towards music, as typically music in this setting is provided by speakers and therefore could have an impact not only on the patient but also on them.

Therefore, we conducted a nationwide survey aiming to evaluate the dissemination of music in the obstetrical setting. The study also considers possible factors impeding the broader use of music in everyday routine among obstetricians and midwives in Germany.

## Materials and methods

Participation was limited to physicians working in obstetrics and to midwives. We identified possible participants by a conclusive nationwide web search, gathering published email addresses on hospitals’ webpages of obstetricians and midwives. An invitation link was sent with the request to participate in the online survey. In total, 2325 invitations were sent to 1763 physicians and 562 midwives working at 730 hospitals in Germany.

We developed a questionnaire comprising the following descriptive data: age, sex, profession and position, federal state, perinatal care level of the department, annual birth rate and number of inpatient beds. We asked to give information about the respondents’ personal relationship towards music (frequency of music consumption, genre, ability to play an instrument and frequency of practice). Then the use of music during caesarean section and vaginal birth was evaluated separately by identical questions regarding the possibility to play music in the respective situation, number of attended births, frequency of music and the process of decision-making. Furthermore, we evaluated the participants’ preferred genre, tempo and volume as well as estimation about the impact of music on communication within the team and with the patient and suspected impact of music on the patient. Finally, we asked whether they already heard or read about positive or negative effects of music on the patient or the team.

Interval scaled items were measured on an analogously 0 to 100 slider scale. Ordinal scaled items were measured on a five-point Likert scale ranging from − 2 to + 2 with 0 being neutral (Table 1).

**Table 1:** Items, scale and response options

Item	Scale	Range
Tempo	Slider scale	0 = "very slow" to 100 = "very fast"
Volume	Slider scale	0 = "hardly audible" to 100 = "very loud"
Assumed impact of music on communication within the team	Likert scale	− 2 = "negative" to + 2 = "positive"
Assumed impact of music on communication with the patient	Likert scale	− 2 = "negative" to + 2 = "positive"
Assumed impact of music on oneself	Likert scale	− 2 = "agitating" to + 2 = "calming"
	Likert scale	− 2 = "distracting" to + 2 = "focusing"
	Likert scale	− 2 = "negative mood" to + 2 = "positive mood"
Assumed impact of music on the patient	Likert scale	− 2 = "agitating" to + 2 = "calming"
	Likert scale	− 2 = "negative mood" to + 2 = "positive mood"
	Likert scale	− 2 = "increases stress" to + 2 = "reduces stress"
	Likert scale	− 2 = "increases anxiety" to + 2 = "reduces anxiety"
	Likert scale	− 2 = "increases pain" to + 2 = "reduces pain"
Use of music in one's spare time	Likert scale	− 2 = "never" to + 2 = "very frequently"

The survey was implemented using https://www.soscisurvey.de/ (SoSci Survey GmbH, Munich, Germany) (a printed version is available as supplemental material). After 21 days, a reminder was sent via email.

### Ethical considerations

All data were acquired anonymously. At the beginning of the survey, participants were informed that answering the survey was completely voluntary and no identifiable data would be collected. It was stated that taking part in the survey was presumed to indicate consent. The study was prospectively approved by the ethics committee of the Heinrich-Heine-University Düsseldorf.

### Statistical analysis

The statistical software package SPSS 24 (IBM Inc., Armonk, NY) was used for all data analyses. Exact numbers and percentage values are presented throughout “Results” section. Group comparisons with dependent variables with interval scales were calculated using independent-sample t-tests. In order to examine the ratings of the effects of music on medical teams and patients, values were tested against neutral with a one-sample *t*-tests. Spearman correlations were used to investigate correlations between interval scaled and categorical variables. Chi-squared tests were applied to examine group differences in categorical dependent variables.

## Results

**Fig. 1:**
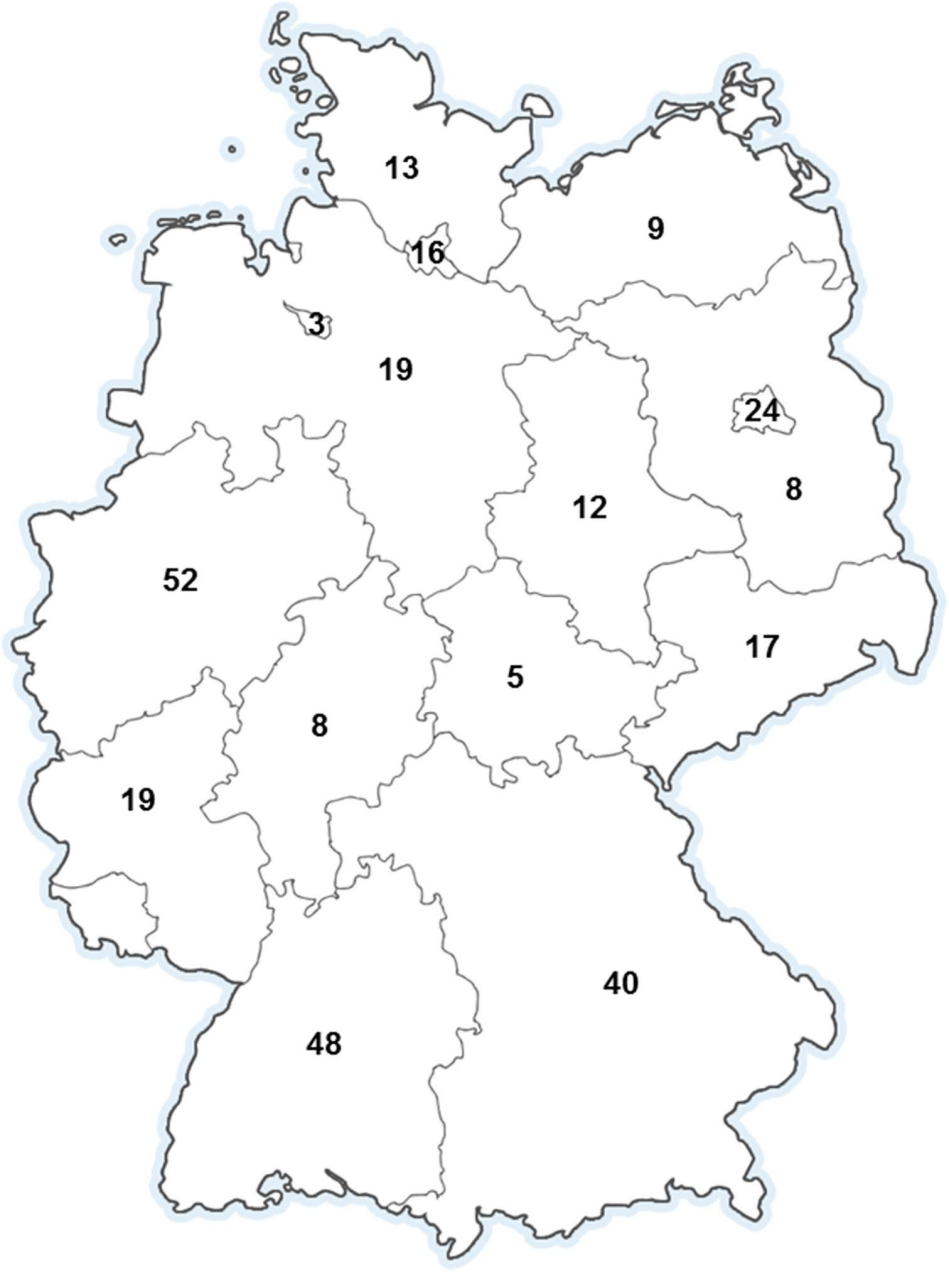
Origin of participants by state

Of all invitees, 180 (10.2%) physicians and 113 (20.1%) midwives completed the survey, totalling 293 professionals. Therefore, the overall response rate was 12.6%. Gynaecologists and midwives from all German states took part in the survey except for Saarland (Fig. 1). Among physicians, 115 (64%) were female, whereas all midwives were female. Physicians and midwives also differed significantly in terms of age, number of accompanied births per year and ability to play an instrument. Table 2 gives an overview of this differences and other demographics and clinical background of the participants.

**Table 2:** Demographics and clinical background of the participants

	Physicians	Midwives	Total	*p*
Total	180 (100%)	113 (100%)	293 (100%)	n.a
Female	115 (64%)	113 (100%)	228 (78%)	< 0.001
Male	65 (36%)	0 (0%)	65 (22%)	
Mean age	42.3	39.2	41.1	0.011
Position				
Chief physician	29 (16%)	n.a		n.a
Senior physician	95 (53%)	n.a		
Specialist	18 (10%)	n.a		
Assistant physician	38 (21%)	n.a		
Level of care				
Perinatal centre level 1	96 (53%)	52 (46%)	148 (51%)	0.054
Perinatal centre level 2	29 (16%)	10 (9%)	39 (13%)	
Basic perinatal care	18 (10%)	18 (16%)	36 (12%)	
No answer	37 (21%)	33 (29%)	70 (24%)	
Annual births per institution				
> 2100	52 (29%)	29 (26%)	81 (28%)	0.100
1800—2100	26 (14%)	11 (10%)	37 (13%)	
1500—1799	19 (11%)	8 (7%)	27 (9%)	
1200—1499	29 (16%)	11 (10%)	40 (14%)	
900—1199	22 (12%)	20 (17%)	42 (14%)	
600—899	16 (9%)	14 (12%)	30 (10%)	
300—599	16 (9%)	19 (17%)	35 (12%)	
< 300	0 (0%)	1 (1%)	1 (0%)	
Annual vaginal births per respondent				
> 150	131 (73%)	45 (40%)	176 (60%)	< 0.001
50—149	39 (22%)	42 (37%)	81 (28%)	
< 50	10 (6%)	26 (23%)	36 (12%)	
Annual caesarean sections per respondent				
> 150	94 (52%)	27 (24%)	121 (41%)	< 0.001
50—149	62 (34%)	21 (19%)	83 (28%)	
< 50	24 (13%)	65 (58%)	89 (31%)	
Ability to play an instrument				
Yes	110 (61%)	49 (43%)	159 (54%)	0.004
No	70 (39%)	64 (57%)	134 (46%)	
Frequency of music listening				
Often or very often	122 (68%)	87 (77%)	209 (71%)	0.111
Occasionally or seldom	58 (32%)	26 (23%)	84 (29%)	

### Music during CS

Of all respondents, 156 (53%) indicated that they do not have the means to play music in the operating theatre and this sub-sample was therefore not further included in this section of the analysis.

One hundred and thirty-seven (47%) stated that they have the technical means to play music in the operating theatre during CS. They indicated that in average they play music in 15% of the cases (range 0–95%). Forty-two (31% of those who have the opportunity to play music) specified that they never play music during CS. The decision whether music is played and which type of music is most often made by the patient (47%), followed by the gynaecologist (37%), nursing staff (8%), anaesthetist (7%) and midwife (2%).

In general, slow music (*M* = 40.1 ± 16.9; *t*(101) = -5.9, *p* < 0.001) played quietly (*M* = 31.9 ± 12.4, *t*(101) = − 14.7, *p* < 0.001) was favoured. Independent-sample t-tests revealed a significant difference between gynaecologists and midwives for the preferred tempo (gynaecologists *M* = 43.0 ± 16.7, midwives *M* = 32.4 ± 15.2, *t*(100) = 2.95, *p* = 0.004), whereas there was no difference for the preferred volume (*p* = 0.222).

Music was estimated to be positive for the communication within the team (*M* = 0.56 ± 1.21; *t*(136) = 5.43, *p* < 0.001) as well as between the medical team and the patient (*M* = 0.62 ± 1.14; *t*(136) = 6.38, *p* < 0.001). The estimations did not differ between professional groups (*p* values > 0.094). There was a positive correlation between the frequency of usage and the estimate of music being positive for the communication in the team (*r* = 0.228, *p* = 0.007). There was no significant correlation between the frequency of usage and the estimate of music being positive for the communication with the patient (*r* = 0.145, *p* = 0.091).

**Fig. 2:**
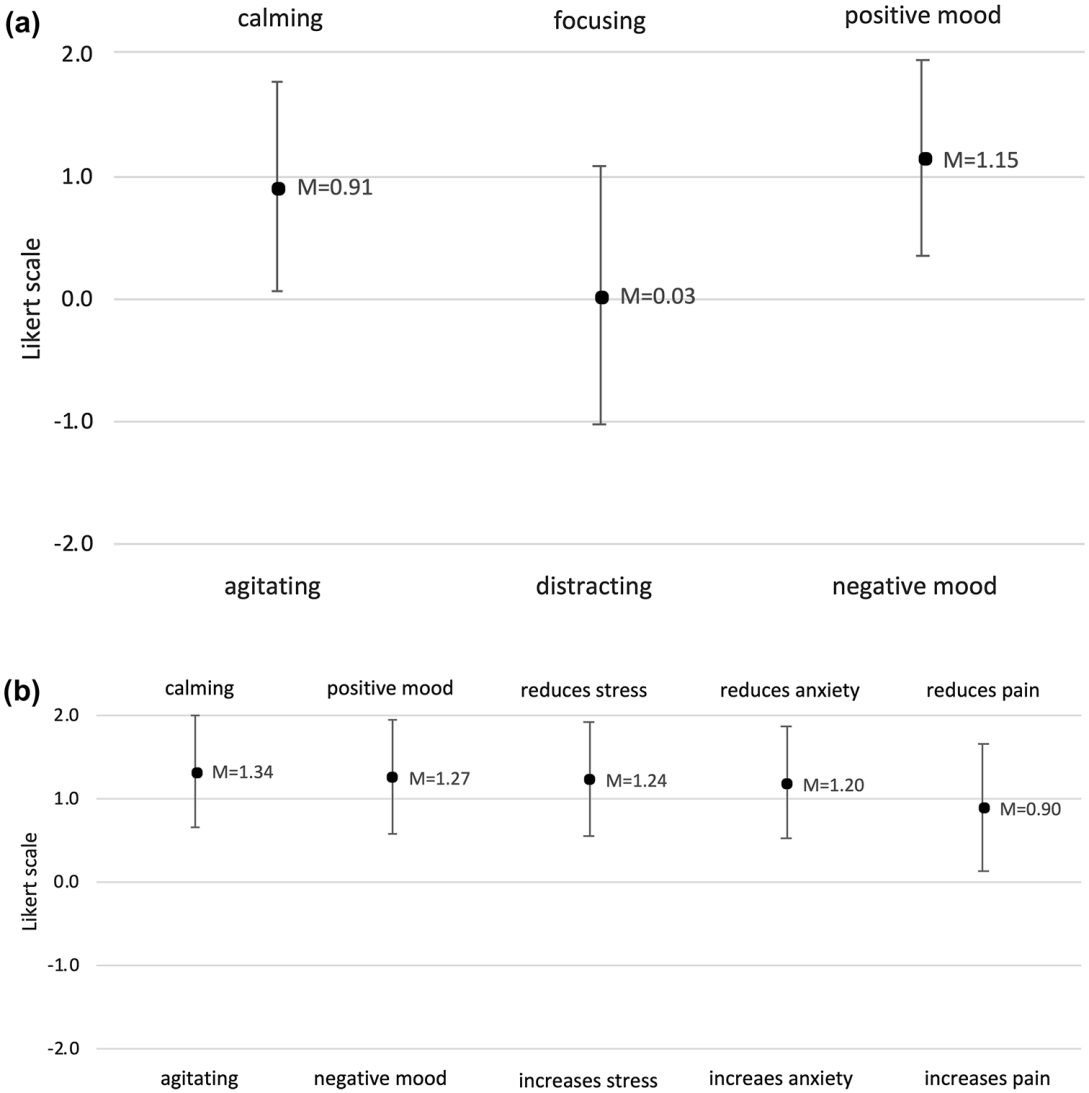
**a** Impact of music on respondents during CS and **b** presumed impact on patients during CS

The respondents stated a calming (*M* = 0.91 ± 0.84, *t*(136) = 12.95, *p* < 0.001) and positively mood changing effect (*M* = 1.15 ± 0.80, *t*(136) = 16.76, *p* < 0.001) of music on themselves. Regarding the question whether music is distracting or helps to focus, the medical team gave neutral responses (*M* = 0.03 ± 1.05; *p* = 0.745; Fig. 2a). There were no differences between midwives and gynaecologists regarding these estimates (all *p*-values > 0.601).

Regarding the impact of music on the patient, survey respondents rated the use of music during CS on all scales as being positive for the patient (Fig. 2b). Again, there were no significant differences between the answers of midwives and gynaecologists (*p* > 0.427).

38% (*n* = 52) of the respondents would recommend listening to music during a CS, whereas 62% (*n* = 85) would not. Midwives would recommend listening to music during a CS significantly more often than gynaecologists [58% vs. 30%, *χ*^2^(137) = 8.88, *p* = 0.003].

### Music during VB

Of 293 respondents, 9 (3%) indicated that they do not have the means to play music in the delivery room and this sub-sample was therefore not further included in this section.

Two hundred and eighty-four (97%) respondents stated that they have the technical means to play music during VB. They indicated that music is played in 38% of cases (range 0–100%). The decision whether and which kind of music is played is made by the patient (94%) or the midwife (6%). Noteworthy, no respondent stated that this decision is made by the obstetrician. Survey respondents favoured slow music (*M* = 38.3 ± 15.7; *t*(252) = −11.83, *p* < 0.001) played softly (*M* = 33.1 ± 12.7, *t*(252) = − 21.2, *p* < 0.001). Independent-sample *t*-tests revealed no significant difference between gynaecologists and midwives for the preferred tempo or volume (*p*-values > 0.473).

Survey respondents indicated that listening to music during VB is positive for the communication within the team (*M* = 0.57 ± 0.97; *t*(283) = 9.89, *p* < 0.001) as well as between the medical team and the mother-to-be (*M* = 0.68 ± 0.96; *t*(283) = 11.84, *p* < 0.001). The estimations did not differ between the professional groups (all *p* > 0.498). There were positive correlations between the frequency of usage and the estimate of music being positive for the communication in the team and with the patient (*r* = 0.202, *p* = 0.001; *r* = 0.152, *p* = 0.010, respectively).

**Fig. 3:**
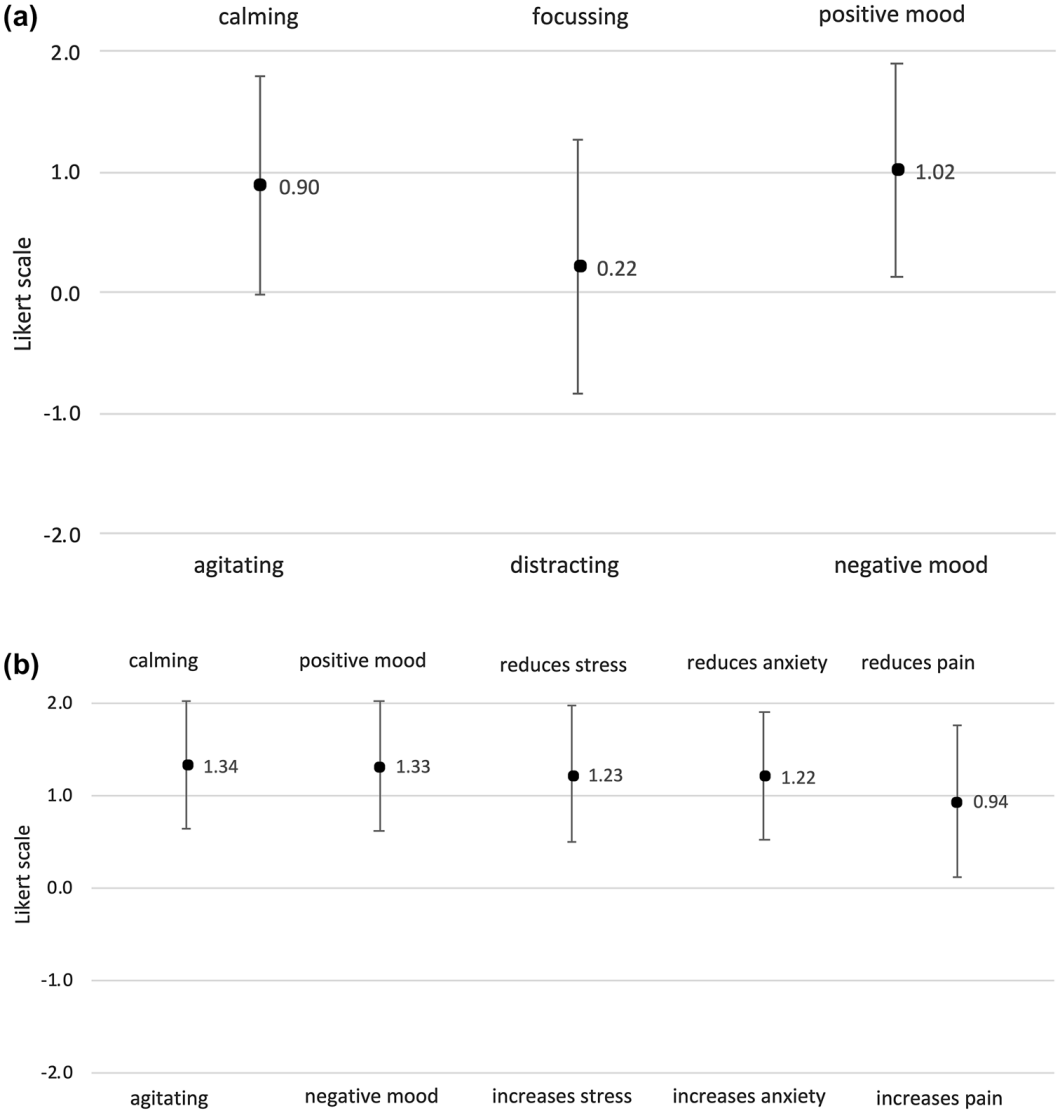
**a** Impact of music on respondents during VB and **b** presumed impact on patients during VB

Respondents stated that during VB music has a calming effect on them (*M* = 0.90 ± 0.91, *t*(287) = 16.83, *p* < 0.001), puts them in a positive mood (*M* = 1.02 ± 0.88, *t*(287) = 19.73, *p* < 0.001) and helps to focus (*M* = 0.22 ± 1.06; *t*(287) = 3.56, *p* = 0.001; Fig. 3a). There were no differences between midwives and gynaecologists (*p*-values > 0.108).

As for the patient, the survey revealed that gynaecologists and midwives rate the use of music during VB in all scales positively (Fig. 3b). Again, there were no significant differences between the estimations of midwives and gynaecologists (*p* > 0.398).

Of the interviewed gynaecologists and midwives, 186 (66%) recommend listening to music during VB, whereas 34% (*n* = 98) do not. A significant relationship between profession and a recommendation could be revealed (*χ*^2^(284) = 23.59, *p* < 0.001). Midwives (83%, *n* = 91) recommend listening to music during VB significantly more often than gynaecologists (55%, *n* = 95). Table 3 summarizes the main items with respect to interprofessional differences.

**Table 3:** Interprofessional comparison of answers

Item	Midwives	Physicians	p value
Preferred tempo during CS	32.4 ± 15.2	43.0 ± 16.7	*0.004
Preferred volume during CS	29.5 ± 12.4	32.9 ± 12.3	0.222
Assumed impact of music on communication within the team during CS	0.842 ± 1.10	0.455 ± 1.24	0.094
Assumed impact of music on communication with the patient during CS	0.842 ± 1.17	0.535 ± 1.12	0.159
Recommendation to listen to music during CS	58%	30%	*0.003
Preferred tempo during VB	40.3 ± 14.7	38.9 ± 16.3	0.473
Preferred volume during VB	33.6 ± 12.5	34.3 ± 12.6	0.688
Assumed impact of music on communication within the team during VB	0.582 ± 0.96	0.558 ± 0.97	0.836
Assumed impact of music on communication with the patient during VB	0.627 ± 0.96	0.707 ± 0.96	0.498
Recommendation to listen to music during VB	83%	55%	* < 0.001

*Statistically significant difference

### Comparing the use of music during CS and VB

The survey revealed that the means to play music are less often available during CS (47%, *n* = 137) than during VB (97%, *n* = 288). Additionally the frequency of music during births in delivery rooms which have the necessary technical equipment is significantly higher during VB (*M* = 42%) than CS (*M* = 15%), *t*(136) = 11.10, *p* < 0.001. Analogously, the medical team would also recommend listening to music to the patient more often during VB (66%) than CS (38%).

**Fig. 4:**
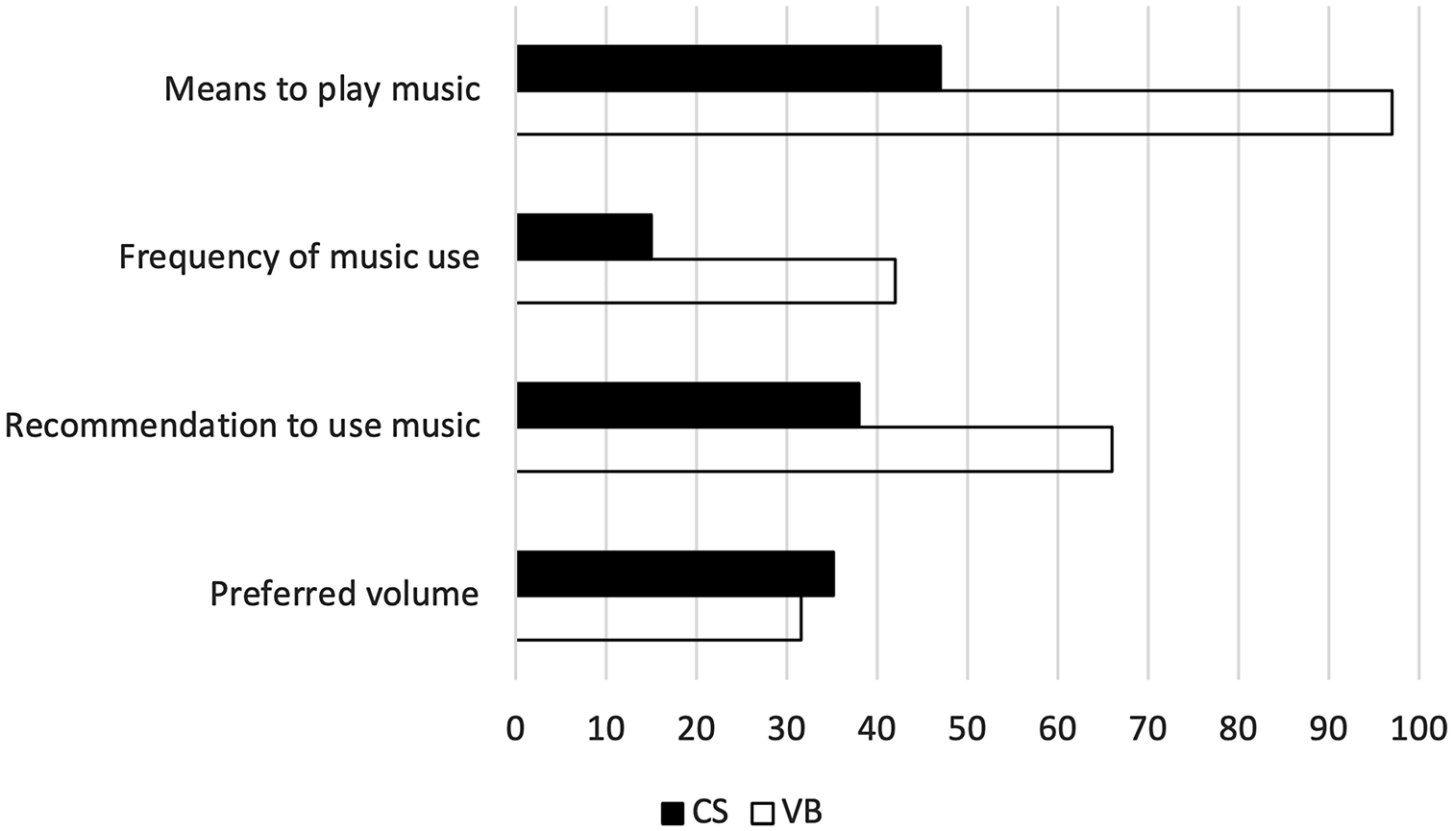
Differences in the use of music during CS and VB

A paired-sample t-test comparing the preferred volume of the music revealed that the respondents indicated to favour music played softer during CS than VB (31.6 vs. 35.2, *t*(95) = 3.63, *p* = 0.006). Whereas music is thought to be neither distracting nor focusing during CS (*M* = 0.03, *p* = 0.745), answers for VB state that it helps to focus (*M* = 0.27; *p* = 0.001). This difference is statistically significant (*t*(136) = 3.30, *p* = 0.001). Figure 4 illustrates the difference in the use of music during CS and VB.

### The influence of participants’ music use in daily life on the use in clinical practice

Of the sample, 209 participants (71%) indicated that they listen to music often or very often in everyday life, whereas 84 (29%) stated that they only listen to music occasionally or seldom. Nobody indicated that they never listen to music. Those who listen to music often or very often recommend the use of music during CS (44% vs. 19%, *χ*^2^(137) = 6.54, *p* = 0.011) and VB (71% vs. 51%, *χ*^2^(288) = 10.13, *p* = 0.001) more often than those who listen to music occasionally or seldom.

Analogously those who listen to music often or very often compared to respondents who listen to music occasionally or seldom tend to rate its impact on team communication more positively during CS (*M* = 0.69 ± 1.20 vs. *M* = 0.15 ± 1.17, *t*(136) = 2.19, *p* = 0.030) and VB (*M* = 0.65 ± 0.99 vs. *M* = 0.34 ± 0.88, *t*(283) = 2.46, *p* = 0.014).

Of all participants, 159 (54%) stated that they learnt to play an instrument. Hundred thirty-nine (47%) still actively play their instrument for an average of 1.4 h per week. The recommendation to listen to music during CS and VB or the ratings on how music influences the communication within the team and with the patient did not differ depending on whether the responded learnt how to play an instrument or not (*p*-values > 0.156).

## Discussion

This nationwide survey sought to evaluate the use of music in obstetrics in German hospitals and to gather information about the attitude of obstetricians and midwives towards music during VB and CS. Among the participants 51% percent work at perinatal care level 1 centres. This indicates that the composition of the sample in this survey seems to be well analogously to the German obstetrical landscape, as 53% of obstetrical departments taking part in a 2017 mandatory federal survey were perinatal care centres level 1 [11]. Furthermore, it indicates that the study represents the reality of a vast amount of births in German hospitals.

Overall the results demonstrate a positive attitude towards music among obstetricians and midwives alike. The respondents report a beneficial influence on the expectant mother and the healthcare providers regardless of the mode of delivery. In spite of other studies indicating a focusing and calming effect of music in the operating theatre on medical staff [12], it seems better implemented in the setting of VB compared to CS. One could speculate that the significant lower availability of music in the operation theatres than in labour rooms (47% vs. 97%) or the rather temporal confinement of the very standardized procedure of CS is responsible for this observation. Nevertheless, our respondents stated also a calming and positively mood changing effect, but showed a neutral position towards a focusing effect during CS. This is in contrast to VB where also a focusing effect is attributed to music.

It is remarkable that professional groups did not differ in their assessment of the effects of music, but in their recommendation regarding its usage during delivery. While obstetricians and midwives alike value the positive effects of music during birth, significantly more midwives recommend its use than obstetricians regardless of the mode of delivery. This seems comparable to the results of another study examining healthcare providers’ musical preferences and views of music in the ambulatory operating room where attending physicians showed significantly lower music enjoyment scores than nurses [13]. Another study showed that the willingness to listen to music is higher among female healthcare professionals than among their male colleagues [7]. As the proportion of male respondents is significantly higher in the obstetricians group, this might also influence their behaviour when it comes to recommendations. It could also partly explain why our study shows a more firmly implementation of music in vaginal births, as gynaecologists are less involved in the decision whether music is played or not (37% in CS vs. 0% in VB).

The survey confirms the presumed positive correlation between healthcare professionals’ attitude towards music and the use of music during CS and VB. It therefore seems important to spread information about research showing that music positively influences several aspects of childbirth such as anxiety, pain, stress and satisfaction regardless of the mode of delivery and can positively affect medical staff [2–5, 10, 14–20], thus overcoming the reluctant use of music and fostering the installation of the necessary means to provide music in obstetrical departments.

### Limitations

A limitation of our study is that the survey only addressed German healthcare providers. It would be interesting to expand the questionnaire about use of music in obstetrics to an international scale in order to compare putative cultural differences. Based on the current results, it would also be highly interesting to evaluate the patients’ attitude to music during childbirth in this context with a large Internet-based survey, thereby also comparing how women plan to use music and the actual use after having given birth.

Another limitation to this study is that it excludes the view of other medical personnel like anaesthesiologists and scrub nurses. Since our survey aimed to include both modes of delivery (CS and VB), we had to exclude professional groups that routinely do not take part in VB. In this context, it should be noted that there are data indicating a detrimental effect of music in operating theatres especially on anaesthesiologists [10, 21].

## Conclusion

This questionnaire-based study shows that music plays an important role during VB. It seems that during one third of VB music is used—mainly upon patient’s request.

In contrast, more than half of the respondents lack the technical means to play music in the operation theatre during CS. And even if possible, music is only rarely used. This is even more noticeable since music was deemed positive in terms of communication, calmness and mood. Therefore, an installation of means to play music as well as ongoing information about its positive impact on patients and professionals alike should be pursued as a target.

## Data Availability

Upon reasonable request to the corresponding author, data are available.
